# Small GTPase Rab21 Mediates Fibronectin Induced Actin Reorganization in *Entamoeba histolytica*: Implications in Pathogen Invasion

**DOI:** 10.1371/journal.ppat.1004666

**Published:** 2015-03-02

**Authors:** Merlyn Emmanuel, Yumiko Saito Nakano, Tomoyoshi Nozaki, Sunando Datta

**Affiliations:** 1 Department of Biological Sciences, Indian Institute of Science Education and Research Bhopal, Bhopal, India; 2 Department of Parasitology, National Institute of Infectious Diseases, Tokyo, Japan; University of Virginia Health System, UNITED STATES

## Abstract

The protozoan parasite *Entamoeba histolytica* causes a wide spectrum of intestinal infections. In severe cases, the trophozoites can breach the mucosal barrier, invade the intestinal epithelium and travel via the portal circulation to the liver, where they cause hepatic abscesses, which can prove fatal if left untreated. The host Extra Cellular Matrix (ECM) plays a crucial role in amoebic invasion by triggering an array of cellular responses in the parasite, including induction of actin rich adhesion structures. Similar actin rich protrusive structures, known as ‘invadosomes’, promote chemotactic migration of the metastatic cancer cells and non-transformed cells by remodeling the ECM. Recent studies showed a central role for Rab GTPases, the master regulators of vesicular trafficking, in biogenesis of invadosomes. Here, we showed that fibronectin, a major host ECM component induced actin remodeling in the parasite in a Rab21 dependent manner. The focalized actin structures formed were reminiscent of the mammalian invadosomes. By using various approaches, such as immunofluorescence confocal microscopy and scanning electron microscopy, along with *in vitro* invasion assay and matrix degradation assay, we show that the fibronectin induced formation of amoebic actin dots depend on the nucleotide status of the GTPase. The ECM components, fibronectin and collagen type I, displayed differential control over the formation of actin dots, with fibronectin positively and collagen type I negatively modulating it. The cell surface adhesion molecule Gal/GalNAc complex was also found to impose additional regulation on this process, which might have implication in collagen type I mediated suppression of actin dots.

## Author Summary

*Entamoeba histolytica* is one of the major causes of morbidity and mortality in developing economies. Severe amoebic infection leads to metastatic spread of the pathogen to extra intestinal sites, especially the liver, causing hepatic abscess. The migratory ability of the pathogen contributes to the spread of the disease. Here, we report that Rab21, a Ras superfamily GTPase, promotes actin dot formation under the fibronectin induced signal in *E*.*histolytica*. The amoebic actin dots share structural components and functional properties to a class of actin rich degradative counterparts found in higher eukaryotes called “invadosomes”. Invadosomes are associated with both normal animal development and diseased state such as cancer. We also show that collagen type I, another major ECM component, suppresses the genesis of actin dots, possibly through interaction with amoebic cell surface adhesion molecule, the Gal/GalNAc lectin complex. Based on our observations, we propose that Rab21 may play an important role in *in vivo* tissue invasion by the parasite which may have further implication in its virulence.

## Introduction

The protozoan parasite, *Entamoeba histolytica*, is the causative agent of amoebic dysentery. Approximately 35–50 million cases of clinical amoebiasis are reported worldwide with 100,000 deaths per year. Despite substantial improvement in the global sanitary facilities (WHO Water Sanitation and Hygiene Data), amoebiasis still poses as a burden on the health care system of the developing economies. The infection can soar up with variable outcomes manifesting in diarrhea, invasive colitis or metastatic infection. The invasive disease pathologies are usually associated with massive destruction of the host tissue. This is caused partly due to invasion of the parasite through the intestinal epithelium and it’s migration to the extra intestinal sites and pro inflammatory responses of the host. Both these factors contribute to amoebic hepatic abscess [1]. Although, amoebiasis poses as a major health risk to the developing countries, the studies reporting molecular basis of tissue invasion are very limited [1].

The actin cytoskeleton organization and dynamics plays an important role in motility related functions of the parasite, many of which are relevant for the observed invasive pathologies. During colonization and infection, the parasite comes in the contact with the host extracellular matrix. The host milieu presents the pathogen with overwhelming stimuli in the form of various extracellular matrix (ECM) components. The ECM cues in turn can activate an array of signaling pathways which lead to remodeling of actin cytoskeleton in the parasite [2]. Furthermore, adhesion to fibronectin (FN), a ubiquitous ECM component, is known to induce secretion of amoebic proteases which locally degrade the ECM and promote random and directed motility of the parasite[3,4]. The fibronectin receptor (β1*Eh*FNR) has been identified and characterized in *E*.*histolytica* and is antigenically similar to the human β1 integrin [5].FN is also shown to induce actin rich dots in *E*.*histolytica*, structures that visually resemble the mammalian invadosomes [6]. Thus, fibronectin act as a potent signal for the parasite, inducing actin remodeling in various ways. Though the involvement of FN in amoebic cytoskeletal remodeling is well presented in the literature, identity of the molecular players acting downstream of fibronectin remains mostly unknown.

Eukaryotic cells constantly converse with their surrounding by forming various actin rich structures. These, prominently include focal adhesion, lamelliopodia, filopodia and invadosomes [7,8]. The invadosomes found exclusively in transformed cells and cells of monocytic origin are formed as a result of an interplay between the structural proteins *viz* vinculin, paxillin and actin regulatory and nucleating factors like Arp2/3 (actin related protein 2/3), N-WASP(Neuronal Wiskott Aldrich syndrome protein), Rho GTPases[9]. These are dynamic structures of rapidly polymerizing actin which are jointly regulated by cues from the ECM and growth factors. Invadosomes allow a cell to integrate remodeling and degradation of the ECM with cell motility [10]. They act as hubs for the activity of the integrins [11,12] and are the major sites for ECM degradation due to focalized secretion of matrix metalloproteinases (MMPs) [13–15].

The importance of Rab GTPases, a class of small G proteins, which principally coordinate vesicular trafficking in eukaryotic cells, has been recently realized in formation and maturation of invadosomes [16,17]. Rabs act as molecular timers for host of cellular processes in a nucleotide dependent manner [18,19].Dysregulated expression of Rab GTPases has been associated with various cancers [20,21]. Change in expression of Rabs could lead to abnormal trafficking of the growth factor receptors, cell surface integrins and matrix metalloproteinases which could further cause increased cell proliferation and migration. Active invadosomes are marked by the presence of MT1MMP (membrane type1 matrix metalloproteinase), a principal membrane anchored collagenolytic protease. Recently Rab5a, Rab8a and Rab14 have been identified as regulators of vesicular trafficking of MT1MMPin mammalian cells [22,23]. While the above subset of Rabs regulate matrix degradation, Rab21 and Rab25 govern cell adhesion and migration by interacting with integrin heterodimers [24,25].

Here, we showed that upon FN stimulation, *Entamoeba histolytica* forms unique actin richdots on its ventral surface in a Rab21 dependent manner.Rab21 could induce *de novo* amoebic actin dots in a nucleotide (GDP/GTP) dependent manner. Interestingly, additional regulation was observed over the process by cell surface adhesion molecule, the Gal/GalNAc (galactose/N-acetyl galactosamine) lectin complex which may have implication in collagen type I mediated suppression of the actin dots. Over expression of Rab21CA (constitutive active or GTP hydrolysis deficient mutant) increased the invasive index of the trophozoites as was evident from the transwell matrigel invasion and fluorescent matrix degradation assays. Taken together, our results indicate that Rab21 along with the host ECM components plays a potential role in tissue invasion and thereby may contribute to the severity of amoebic infection.

## Results

### The Extracellular matrix of the host can induce actin dots in *Entamoeba histolytica*

*E*.*histolytica* has a dynamic cytoskeleton, allowing it to respond to the signaling inputs from the environment. Host ECM forms an intricate part of the parasite surroundings. Therefore we wanted to study the effect of ECM components on the parasite’s cytoskeleton.

**Fig 1:**
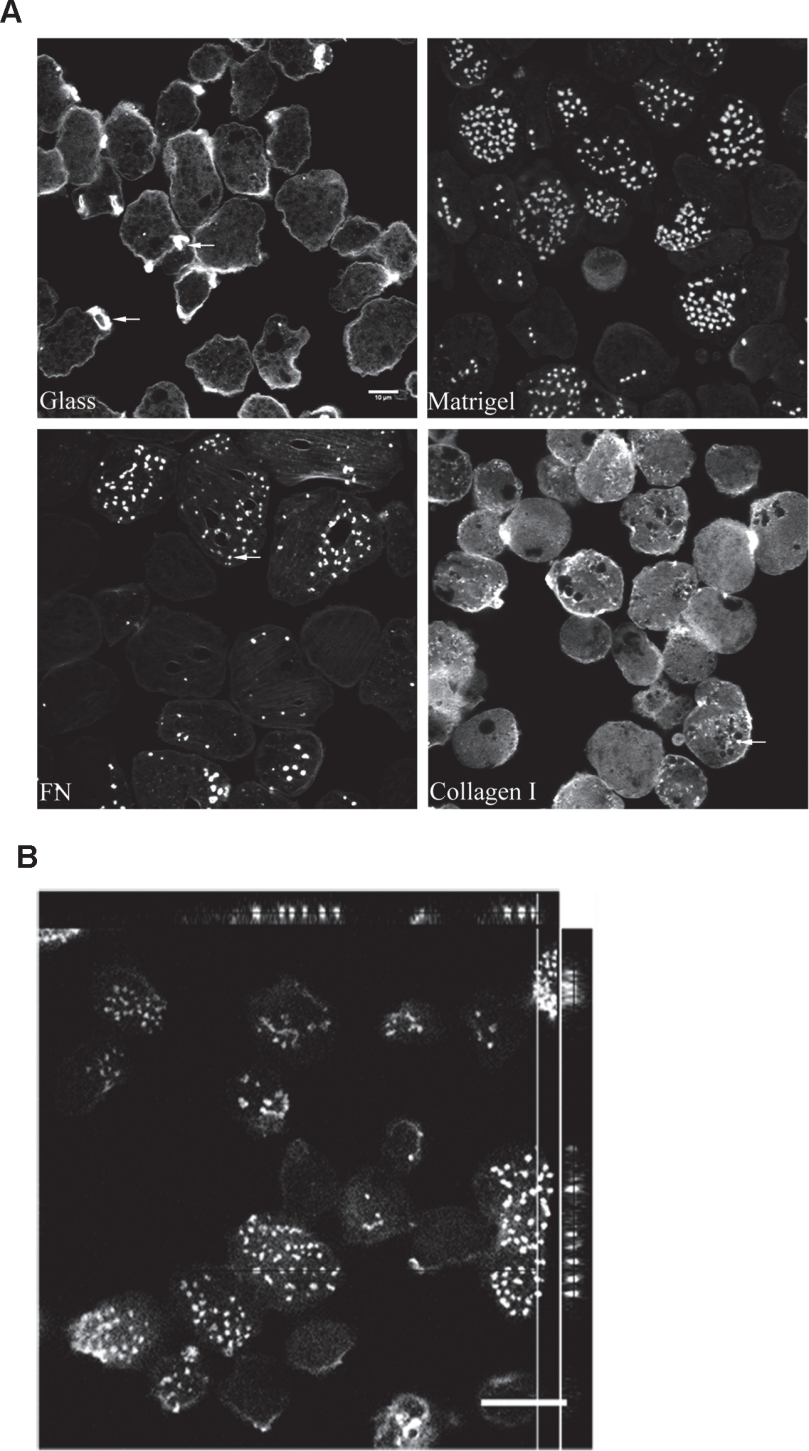
Host ECM components induces actin rearrangement in *Entamoeba histolytica*. **A.** Control trophozoites were incubated for an hour on Glass and various ECM coated surfaces; Matrigel (100μg/ml); Fibronectin (100μg/ml) and Collagen type I (100μg/ml) at 35°C. Cells were fixed and stained for actin using Alexa568 Phalloidin and imaged using Zeiss LSM 780. **B.** An orthogonal view of the cells forming actin dots with mean area of 2.5±0.21μm^2^ and a depth of 2–3μm. A representative xy section together with selected xz and yz is shown. For calculation of mean area of actin dots formed in presence of FN, a total of 140 actin dots spread over 70 cells were analyzed. Scale bars, 10μm.

Matrigel is a reconstituted basement membrane preparation of heterogeneous composition, an ideal mimic for the host ECM [26].On the other hand, fibronectin is one of the most ubiquitous and abundant ECM proteins. We used matrigel and fibronectin to seek for changes in the actin cytoskeleton of the parasite. When stimulated with matrigel or fibronectin, more than 60% of the total trophozoites formed focalized actin rich dots on the substratum contacting side (Fig. 1A), referred now onwards as amoebic actin dots. These dots had a mean area of 2.5±0.21μm^2^ and extended over 2–3μm in depth (Fig. 1B, orthogonal view of actin dots).We observed a minimum of 2 actin dots per cell to maximum of 42 actin dots per cell with a modal value (N_mode_) of 10 for fibronectin coated surface. In case of matrigel coated surface, we observed a minimum of 3 actin dots per cell to maximum of 40 actin dots per cell with a modal value (N_mode_) of 6.

The actin dots visually resembled ECM degradative structures, the invadosomes [27].This intrigued us to carry out immunostaining for an invadosomal marker, vinculin. Vinculin, an actin binding protein has been previously reported to be a part of the signaling complex assembled in response to the FN exposure in the pathogen [28]. The co-localization of vinculin and actin (S1 Fig.), suggest that the amoebic actin dots also share architectural molecules with mammalian invadosomes.

Since collagen type I, the other major ECM component is known to induce a novel class of linear invadosomes in mammalian cells [29], we further investigated its effect on the actin cytoskeleton of the parasite. Interestingly, unlike fibronectin and matrigel, collagen type I did not promote the formation of actin dots. The actin cystoskeleton remained largely unperturbed in the presence of collagen type I (Fig. 1A).

Invadosomes have a dynamic F- actin core with a rapid turnover [9].Cytochalasin D is a drug widely used against actin polymerization which inhibits the process by binding to the barbed ends of a growing actin polymer. Interestingly, it has also been shown to promote invadopodia formation through activation of Tks5 (tyrosine kinase substrate with five SH3 domains), a Src substrate with a scaffolding role in the assembly and organization of invadopodia [9,30].Thus, the action of cytochalasin D on actin polymerization, especially on invadopodia is counter intuitive. To test how the drug modulates the structure of the amoebic actin dots, we treated the FN stimulated trophozoites with increasing concentrations of cytochalasin D and stained for actin. The drug, at increasing concentration clearly abrogated the morphology of actin rich structures. The most dramatic effect was observed at 10μM of cytochalasin D, where 90–95% of cells were marked by the presence of a long actin thread with bulbous projections at regular intervals (S2 Fig.), suggestive of either a hyper polymerization or lack of depolymerization of actin filaments.

### An over-expression study identifies a subset of Rab GTPases as regulators of actin dots

Rab GTPases, member of Ras superfamily, are master regulators of vesicular trafficking. They control the route traversed by the cargo inside the eukaryotic cells. They also regulate the function of invadosomes by controlling the trafficking of matrix metalloproteinases [22,23,31]. Here, we tested a subset of Rab GTPases *viz*Rab5, Rab21, Rab7A and Rab7B, some of the amoebic homologues of mammalian endocytic Rabs, to decipher their role in amoebic actin dot formation. We also included amoebic homologue of the recycling Rab i.e. Rab11B.

**Fig 2:**
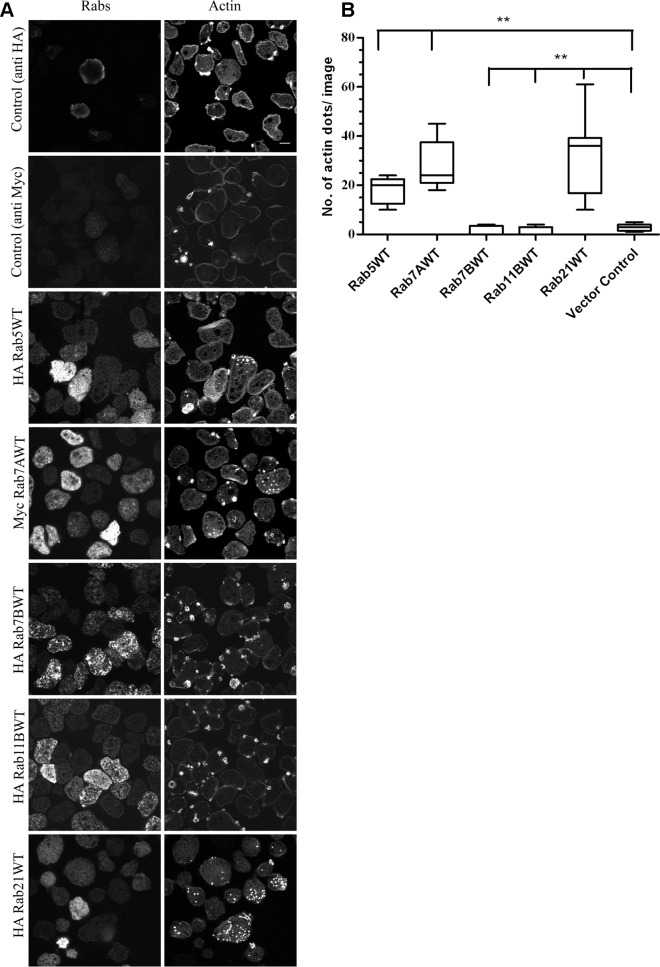
An over-expression study to identify Rabs for their role in biogenesis of amoebic actin dots. **A.** Trophozoites stably expressing HA tagged Rab5, Rab7B, Rab11B, Rab21and Myc tagged Rab7A were plated on uncoated glass surface, fixed and stained for the respective Rabs using mouse MAbs against HA and Myc and for actin using Alexa568 Phalloidin. zstacks (z interval = 1μm) were acquired using Zeiss LSM780. A representative slice from the zstack is shown for all the Rabs that were screened. **B.** Quantification of the number of actin dots formed in various Rab expressing cell lines (n = 300 cells/ cell line).Graph shows mean ± SEM of three independent experiments.**, P<0.01. Scale bars, 10μm.

Stable amoebic transformants of the candidate Rab GTPases and vector control (transfected with pEhExHA/pEhExMyc) were generated. The amoebic cysteine synthase promoter was used for expressing the genes of interest. The stable transfectants were selected and maintained at 20μg/ml G418.The different Rab expressing cell lines were plated on glass coverslips, incubated at 37°C for an hour to attach and stained with Alexa 568Phalloidin for visualizing actin. As shown in Fig. 2A, over-expression of Rab5, Rab7A and Rab21 induced *de novo* formation of actin dots on glass surface. Interestingly, the actin dots were observed in the moderately expressing trophozoites which accounted for 25–30% of the total trophozoites. For calculating the number of actin dots formed in a given cell line, we analyzed approximately 300 trophozoites over 15 images with 20–22 trophozites per image. The quantification for the same has been depicted in Fig. 2B. Interestingly, we observed actin rich adhesion plates for vector control (pEhExHA), Rab7B and Rab11B expressing trophozoites.

### Rab21 acts as a novel regulator of the amoebic actin dots

Although, Rab5, Rab7A and Rab21 induced actin formation in the parasite (Fig. 2A), the primary knowledge on the role of human Rab21 (hRab21)being involved in integrin dependent cell migration and adhesion [24] and complete lack of information of the cellular functions of amoebicRab21 prompted us to continue further studies with Rab21.

Receptor-mediated endocytosis and macropinocytosis are essential cellular functions required for nutrient acquisition. hRab21 regulates both these processes [32].Therefore, to determine whether amoebicRab21 is involved in endocytosis, we carried out classical cargo uptake experiments(manuscript under review,PNTD-D-14-01300).The endocytosis of holo-transferrin and dextran were assayed in trophozoites expressing wild type Rab21 (Rab21WT), constitutive active or GTP hydrolysis deficient mutant (Rab21CA) and dominant negative or GTP binding deficient mutant (Rab21DN) using flow cytometery and confocal microscopy. We did not observe any co-localization betweenRab21 and transferrin or dextran positive compartments. Moreover, over-expression of wild type or mutant Rab21 did not show any detectable effect on the uptake of these cargos.

Phagocytosis plays an important role in nutrient uptake by *E*. *histolytica* and hence is important for its survival and virulence. Rab5 is an important regulator of erythrophagocytosis in amoeba. Since, Rab21 is a member of the Rab5 subfamily, we decided to study whether it is also involved in erythrophagocytosis [33].Cell tracker labeled RBCs were used to measure the phagocytic efficiency of the trophozoites expressing Rab21WT and mutants (S2 Method). As shown in S3 Fig., we did not observe any effect of over-expression of Rab21 on RBC phagocytosis.

**Fig 3:**
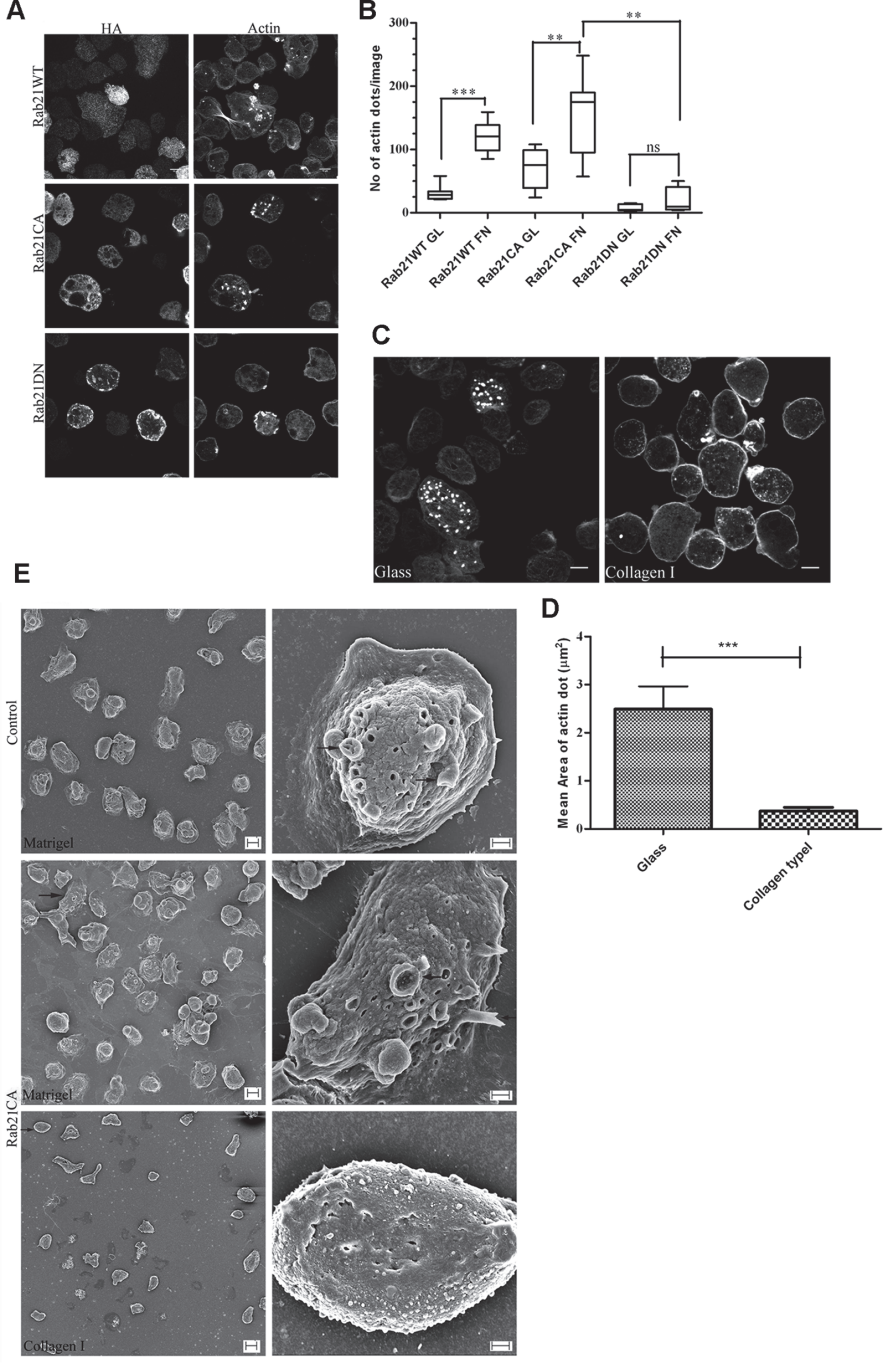
Rab21 regulates the amoebic actin dots. **A.** Trophozoites stably expressing HA tagged Rab21WT, Rab21CA and Rab21DN were harvested and plated on uncoated glass surface, fixed and stained using anti HA, followed by secondary anti mouse Alexa488 and Alexa568 Phalloidin and z stacks were acquired (z step = 1μm). A representative slice from the z stack is shown. Scale bar, 10μm. **B.** Quantification of number of actin dots formed in Rab21WT and CA, DN mutants when plated on glass or FN coated (100μg/ml) coverslips (n = 150cells). Graph shows mean ± SEM of three independent experiments. **, P<0.01.**C.**Trophozoites stably expressing HA tagged Rab21CA were harvested and plated on glass and collagen type I (100μg/ml) coated surface and incubated for an hour at 37°C. Cells were fixed and stained for actin using Alexa568 Phalloidin and z stacks were acquired for every z step of 1μm. A representative slice from the z stack is shown. Scale bar, 10μm. **D.** Quantification of the mean area of actin dots formed in the Rab21CA mutant plated on collagen type I (100μg/ml) coated surface. Mean area was calculated for n = 180 actin dots for collagen type I and n = 145 actin dots for glass surface. Mean diameter was manually measured using the motion tracking software and area calculated, treating the actin dot to be a circular object ($Α=\pi {\text{r}}^{2}$) and plotted using GraphPad Prism5. Graph shows mean ± SEM.***, P<0.001. **E.** Trophozoites transfected with Rab21CA were placed between two layers of either Matrigel at 1mg/ml or collagen type I at 2mg/ml and incubated for 6–8hrs at 37°C. Trophozoites transfected with empty vector were also sandwiched between matrigel as control. Samples were fixed and processed for SEM and imaged using Zeiss Ultra PLUS. Left panel shows lower magnification micrographs (1000X for matrigel, scale bar 10μm and 500X for collagen type I, scale bar 20μm) while the right panel shows higher magnification micrographs (8000X, scale bar 2μm).Arrows indicate surface protrusions produced on Rab21CA cells in a matrigel sandwich compared to the rough surface in a collagen type I sandwich.

In the current study, we observed that over-expression of Rab21 lead to amoebic actin dot formation. Moreover, these actin rich structures were also formed in the presence of ECM, specifically fibronectin. Therefore to further understand whether Rab21 is involved in this ECM mediated process, we made use of the CA and DN mutants of Rab21.These mutants are widely accepted tools to understand whether a cellular process is driven by the nucleotide state of the GTPase. As shown in Fig. 3A, the wild type and CA mutant of Rab21 formed actin dots whereas Rab21DN did not. Further we checked for the effect of fibronectin stimulation on the Rab21WT and mutant expressing trophozoites. As shown in Fig. 3B, the effect of Rab21WT and CA on the actin cystoskeleton was enhanced in presence of the stimuli as compared to unstimulated trophozites. Therefore, based on our observations we suggest that the fibronectin mediated actin rearrangement involves Rab21 and it further depends on the nucleotide bound state (GDP/GTP) of the GTPase.

We further extended our study to investigate the effect of collagen type I, the other major ECM component, on the actin cytoskeleton of the Rab21CA transformants. In contrast to fibronectin, collagen type I suppressed the actin dot formation in Rab21CA transformants (Fig. 3C). Interestingly, smaller actin dots were observed (mean area = 0.35±0.024μm^2^, n = 180 actin dots, 70 cells, Fig. 3D) in comparison to the glass surface (mean area = 2.47±0.2μm^2^, n = 142 actin dots, 50 cells, Fig. 3D).Based on the opposing effect observed for the ECM components, we propose the possibility of differential regulation of the actin cytoskeleton.

To further characterize the structures and to study the surface topology of the trophozoites at high resolution, we did scanning electron microscopy on the Rab21CA expressing trophozoites sandwiched between thin layers of matrigel. The rational for the sandwich culture was to mimic the 3D environment present inside the host upon infection and to induce the protrusive actin rich structures on the dorsal surface of the trophozoites. The trophozoites were incubated for 6–8hrs in the matrigel sandwich and subsequently processed for acquiring images. As shown in the Fig. 3E, Rab21CA upon incubation in the matrigel sandwich formed numerous surface projections. Similar surface morphology was also observed for the vector control (pEhExHA) trophozoites (Fig. 3E, upper panel). In contrast, Rab21CA trophozoites when sandwiched between layers of collagen type I did not form any such protrusive structures but rather had a rough surface devoid of any membrane extensions (Fig. 3E, lower panel).

We also carried out an ultrastructural analysis of the trophozoites expressing Rab21CA and DN mutants along with vector control trophozoites plated on glass. Expression of both the mutants resulted in an overall smooth surface. Interestingly, Rab21CA was well spread out and attached to the glass surface (S4 Fig., middle panel), whereas Rab21DN had a rounded morphology and failed to attach (S4 Fig., lower panel).

### Rab21 modulates matrix degradation and invasion through the matrigel barrier

Metastatic tumor cells can invade and migrate through the matrigel barrier by secreting matrix metalloproteinases [26,34]. The widely used Transwell Matrigel Invasion Assay utilizes the above property to quantify the invasive capacity of the metastatic cells.

**Fig 4:**
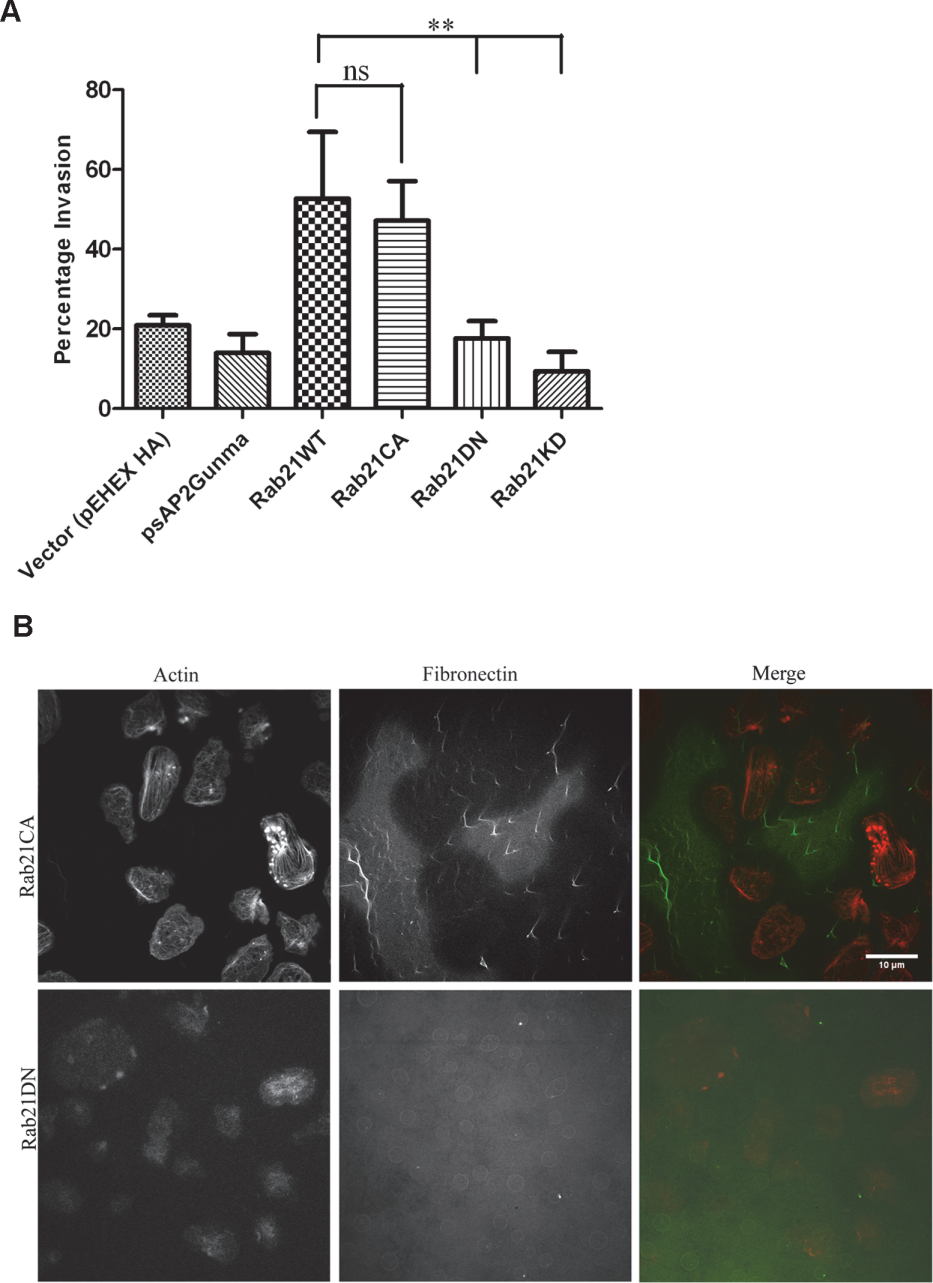
Rab21CA over-expression is associated with increased invasion and matrix degradation. **A.** Trophozoites stably expressing Rab21WT, Rab21KD, Rab21CA and Rab21DN and vector control (pEhExHA for Rab21WT and mutants; psAP2Gunma for Rab21KD strain) were harvested, washed and resuspended in serum free BI medium and 7.5 x10^5^ cells were plated in upper chamber of a transwell insert coated with 5mg/ml of matrigel. The cells were attracted in the lower chamber using 15% adult bovine serum as a chemoattractant. Trophozoites were allowed to invade for 48–50hrs.After 48hrs, the migrated cells in the lower chamber were detached and counted. Graph shows mean ± SEM of three independent experiments. **B.** Trophozoites stably expressing Rab21CA and Rab21DN were plated on glass coverslips coated with Hilyte488 Fibronectin and incubated at 35°C for 45–48 hrs under dark. After 48hrs, the cells were fixed and stained with Alexa568 Phalloidin for actin and confocal images were acquired using Zeiss LSM780. Scale bars, 10μm.

To understand the functional significance of Rab21 induced actin dots in Entamoeba *histolytica* we carried out the invasion assay for Rab21WT, Rab21CA, Rab21DN as well as the knock down strain of Rab21 (Rab21KD). Rab21KD was generated using G3 trophozoites transfected with silencing plasmid psAP2Gunma Rab21 (S1 Method) [35].We observed86.6% reduction in the expression level of Rab21 in the Rab21KD strain as compared to the vector control (pSAP2Gunma) transfected G3 trophozoites (S5 Fig.). Cells were plated in the upper well in serum free medium with the lower well containing adult bovine serum as a chemoattractant. Forty eight hours post-incubation, cells that had invaded were detached from the lower well, harvested and counted. The Rab21CA expressing cells showed increased migration with 47.2±8.6% of the total cells plated in the upper chamber invading through the matrigel barrier outperforming the Rab21DN which showed 17.6±3.7% invasion similar to vector control (21.6±2.6%) whereas the Rab21WT showed 52.7±11.8% invasion in similar setup (Fig. 4A). The Rab21KD showed least invasion with only 9.4±3.4% of total cells invading through the matrigel. The vector control for the Rab21KD strain (psAP2Gunma transfected trophozoites) showed 14.0±3.8% invasion. The above results suggest that Rab21modulates the process of migration and invasion.

Though matrigel invasion proves to be a useful assay for measuring invasive capacity of the cells, the possibility of amoeboid movement through the barrier cannot be ruled out. Therefore, to confirm that the observed matrix invasion was due to active protease secretion, we carried out fluorescent matrix degradation assay. The surface degradation of Hilyte488 Fibronectin was analyzed with time. Cells expressing Rab21CA and DN were plated on Hilyte488 Fibronectin coated coverslips and incubated for 48hrs under dark, after which they were fixed and stained for actin. The Rab21CA expressing trophozoites showed heavy degradation of the fluorescent matrix, with cells showing a halo (i.e. loss of fluorescence) around them (Fig. 4B). Whereas, no matrix degradation was observed for the Rab21DN mutant indicating that actin dots formed as a consequence of the Rab21CA over-expression conferred the cells with increased degradative capacity.

### The surface adhesion molecule Gal/GalNAc complex regulates actin dot formation

Invasion through the matrix barrier relies on the successful attachment of the parasite to the host ECM components. The binding to the host glycoproteins is mediated by the Gal/GalNAc lectin complex, a major amoebic surface receptor [36]. Interestingly, the complex also mediates binding of the parasite to collagen type I [37,38]. Therefore, we decided to study the role of Hgl on biogenesis of the actin dots.

**Fig 5:**
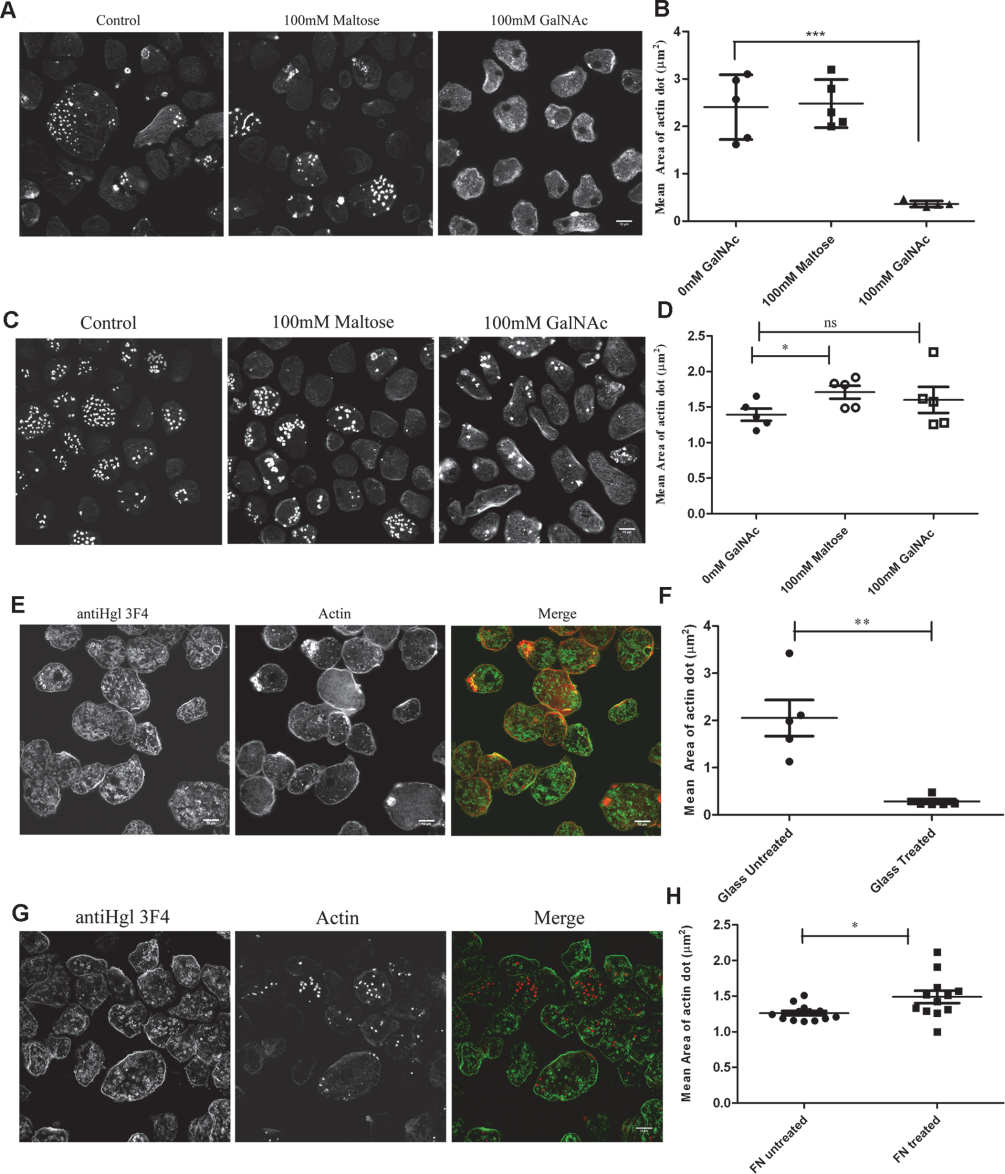
Gal/GalNAc lectin complex inhibits actin dot formation in Rab21CA. Trophozoites stably expressing Rab21CA were incubated for an hour with **A.** GalNAc on glass and fixed and stained for actin. **B.** Quantification of mean area of actin dots in Rab21CA formed on glass surface in presence of 100mM maltose and 100mM GalNAc. Mean diameter was manually measured using the motion tracking software (n = 150 actin dots/condition, total cells counted = 100) and area calculated, assuming the actin dot to be a circular object $Α=\pi {\text{r}}^{2}$) and plotted using GraphPad Prism5. Graph shows mean ± SEM.***, P<0.001.**C.** Rab21 CA transformants were incubated on FN coated surface along with GalNAc for an hour at 35°C. Cells were fixed and stained for actin. **D.** Quantification of mean area of actin dots in Rab21CA formed on FN coated surface in presence of 100mM maltose and 100mM GalNAc (n = 690 actin dots/ 150 cells for 0mM GalNAc; n = 441 actin dots/ 180 cells for 100mM GalNAc; n = 700actin dots/ 220 cells for 100mM Maltose).Graph shows mean ± SEM.*, P<0.05. **E.** Similarly, trophozoites stably expressing Rab21CA were incubated with αHgl mouse MAb 3F4 (1:30) plated on glass for an hour at 35°C. Cells were fixed and stained for Hgl (green) and actin (red) and imaged using Zeiss LSM780. **F.** Quantification of mean area of actin dots in Rab21CA formed on glass surface in presence of αHgl 3F4 (n = 155 actin dots, 91 cells for treated with 3F4; n = 200actin dots, 100cells for untreated).Graph shows mean ± SEM. **, P<0.01. **G.** Trophozoites stably expressing Rab21CA were incubated with αHgl mouse MAb 3F4 (1:30) plated on fibronectin coated surface for an hour at 35°C. Cells were fixed and stained for Hgl (green) and actin (red) and imaged using Zeiss LSM780. **H.** Quantification of mean area of actin dots formed on fibronectin coated surface in presence of αHgl 3F4 (n = 590 actin dots, 105 cells for treated with 3F4; n = 450 actin dots, 98 cells for untreated). Graph shows mean ± SEM. *, P<0.05. Scale bars, 10μm.

The essential role of Carbohydrate Recognition Domain (CRD) of heavy subunit (hgl) of the lectin complex in adherence and cytolysis of target cells has been demonstrated by carbohydrate and monoclonal antibody mediated inhibition assays [36,39].Therefore to study the role of Hgl in biogenesis of actin dots, we blocked the ligand binding site with increasing concentration of GalNAc or monoclonal antibody, αHgl 3F4, raised against the recombinant CRD [39]. The monosaccharide, GalNAc showed an inhibitory effect. As shown in Fig. 5A and 5B,cells expressing Rab21CA mutant when plated on glass showed a dramatic decrease in the number and the size of the actin dots in the presence of GalNAc (mean area on glass = 2.41±0.36μm^2^, n = 95 actin dots, 70 cells; mean area in presence of 100mM GalNAc = 0.36±0.183μm^2^, n = 130 actin dots, 94 cells). In agreement with the GalNAc inhibition assay performed using sugar, the αHgl3F4 antibody also suppressed the formation of the dots on the glass surface {mean area = 2.05±0.15μm^2^ (untreated), n = 283 actin dots, 152 cells; mean area = 0.28±0.05μm^2^ (treated with αHgl 3F4), n = 155 actin dots, 91 cells} (Fig. 5E and 5F).

We further extended our assay to the fibronectin coated surface using both, the sugar and the αHgl 3F4. Interestingly, the inhibitory effect of GalNAc and αHgl 3F4 on actin dots was not observed on fibronectin coated surface (Fig. 5C and 5G). In presence of GalNAc, Rab21CA expressing trophozoites plated on FN coated surface formed actin dots {mean area on FN coated surface = 1.38±0.15 μm^2^, n = 130 actin dots, 60 cells; mean area in presence of 100mM GalNAc = 1.59±0.20 μm^2^, n = 138 actin dots, 65 cells; Fig. 5D}.Similarly, we observed no suppression of actin dots on FN coated surface in presence of αHgl 3F4 {mean area = 1.20±0.30 μm^2^ (untreated), n = 184 actin dots, 134 cells; mean area = 1.48±0.36 μm^2^ (treated with αHgl), n = 130 actin dots, 90 cells; Fig. 5H}.

Based on our above observations, we speculate that the lectin complex *via* its CRD region regulates the biogenesis of actin dots in the parasite.

## Discussion

*Entamoeba histolytica*, a professional phagocyte displays varying responses to the extra cellular matrix (ECM). Interaction with the ECM is important for amoebic invasion of the host tissue and therefore is a key step in the pathogenesis. Both, host and pathogen factors contribute to the invasion process [40,41]. The ECM is a highly diverse and dynamic protein network, providing structural support and signaling cues which regulate cell behavior [42,43]. The cell responds to these signaling inputs by employing various actin rich structures [7]. Invadosomes are a class of actin rich protrusive structures which degrade the underlying matrix by secreting a battery of zinc regulated matrix metalloproteinases (MMPs) [10,27]. Invadosome dependent cell invasion is often seen in cancers but it is also observed during normal animal development[44]and angiogenesis as well as in immune surveillance by leukocytes [45]. Here, we demonstrate that upon exposure to ECM, the protozoan parasite forms similar structures in a Rab21 dependent manner.

The host ECM is a blend of various proteins with fibronectin (FN) and collagen being the major constituents. The physical, topological and biochemical composition of the ECM is highly tissue specific and heterogeneous, varying even within the same tissue and with age of the animal [42,43]. In mammals, adhesion to FN is mediated by the α5β1 integrin heterodimer [25] and similar receptors are thought to exist in *Entamoeba histolytica*. At least two amoebic FN binding proteins have been identified: a 37kDa receptor and a 140kDa receptor (β1*Eh*FNR). β1*Eh*FNR has been shown to have antigenic similarities to the mammalianβ1 integrin [5].They are thought to regulate the binding of the pathogen to the host ECM[5,46]. Binding of the β1*EhFNR* to fibronectin leads to phosphorylation of several proteins, including FAK (focal adhesion kinase), paxillin and vinculin [28]. Fibronectin can also induce major actin rearrangement in *E*. *histolytica* through Rho1 and ROCK dependent pathway[6]. Here, we showed that it promotes the formation of focalized actin dots in the parasite in a Rab21 dependent manner (Fig. 2A).In contrast, the other major ECM component, collagen typeI suppresses the Rab21mediated actin dot formation (Fig. 3C and 3D).The molecular details for the process would require further in depth studies. The possibility of both the ECM components triggering different signaling pathways in the pathogen cannot be overlooked. Thus, the variable ECM composition between individuals may have an implication in their relative susceptibility towards amoebic infection.

Binding of GalNAc and MAb αHgl 3F4 also lead to suppression of Rab21 induced actin dots, in a similar manner as observed for collagen type I. Collagen type I is a bonafide ligand for the Gal/GalNAc lectin complex [37,38].Therefore, based on our observations we propose that the inhibitory effect of collagen type I may be mediated via its ability to bind to the CRD of the Hgl subunit. Possibly, the binding of ligands including collagen type I to the Hgl leads to conformational changes which in turn lead to suppression of actin dots. Interestingly, in the past αHgl3F4 has been shown to enhance binding of the parasite to the host glycoproteins and mucin [39]; in our current experimental setup, effect of αHgl 3F4 on adhesion of the parasite to collagen type I and FN coated surface is not completely understood. Moreover, the Hgl mediated inhibition was not observed on the FN coated surface. The possibility of the collagen receptor being involved in the inhibition process cannot be overlooked as well [47].

In the recent past, Rab GTPases have taken centre stage as regulators of invadopodia in the mammalian cells, governing the trafficking of the matrix metalloproteinases [22,23,31,48]. They both, directly or indirectly, associate with different integrin heterodimers and spatially control their distribution along the migratory axis of the cell [12,24,25].hRab5, the principal early endocytic Rab, was shown to regulate formation and maturation of invadosome [23,48]. hRab21has been reported to regulate cell adhesion and migration in a β1 integrin dependent fashion [24].hRab21 was also shown to regulatematrix remodeling by Cancer Associated Fibroblast (CAFs) and thereby augmenting cell invasion [49].Although hRab21 has been shown to be associated with cancer cell migration and invasion, its role in formation or maturation of invadosomes is yet to be investigated.

*Entamoeba histolytica* has an elaborate network of vesicular trafficking as reflected by the presence of more than 90 members of Rab family GTPases [50]. Till date, only few of Rab proteins have been characterized.Rab5 and Rab7A are known to regulate erythrophagocytosis [33].Additionally, Rab7A is also involved in retromer dependent recycling of the hydrolase receptor [51].Rab7A and Rab7B are also shown to jointly regulate the biogenesis of the phagolysosomal compartment in the parasite [52]. Rab11B plays a central role in the secretion of cysteine proteases, a major virulence factor of the parasite [53]. But like most other amoebic Rab family members the biological function of Rab21 still remains unknown.AmoebicRab21 unlike its mammalian homologue did not show any effect on the uptake of classical endocytic or phagocytic cargos (manuscript under review,PNTD-D-14-01300).In this study, we showed that Rab21acts as a downstream cue under the FN stimulated cytoskeletal remodeling in the parasite. The possibility of Rab21 regulating the trafficking of the *Eh*FNR cannot be ruled out. In a previous report [46], it was shown that FN stimulates protease secretion by the parasite which was further proposed to alter its pathogenic behavior. Here, in the current study, we demonstrated that the ECM degradative activity associated with the actin rearrangement is governed by Rab21 in a nucleotide (GDP/GTP) dependent manner (Fig. 4A and 4B). Therefore, based on our observations, we hypothesize that Rab21 may lead to an overall increase in the proteolytic activity, thereby conferring an advantage of enhanced invasive capacity to the parasite.

**Fig 6:**
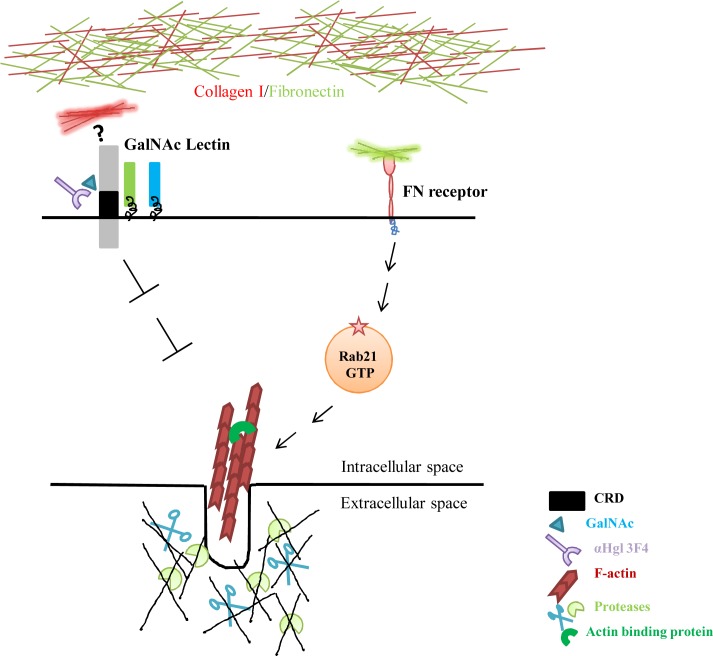
Schematic representation of regulation of amoebic actin dots. The *Eh*FNR bound to the fibronectin, turns on an intracellular signaling cascade. Rab21 acts downstream of the *Eh*FNR, possibly by sensing the ligated form of the receptor, a function dependent on its nucleotide bound state. These signaling events finally lead to changes in the actin cytoskeleton of the pathogen. Collagen type I possibly act through the CRD region of the Hgl subunit of the Gal/GalNAc lectin complex and inhibits the amoebic actin dots.

Taken together, our results show that fibronectin induces actin dots in *E*.*histolytica* in a Rab21 dependent process while collagen type I suppresses the process perhaps in Hgl dependent manner (Fig. 6). Further, the amoebic actin dots were found to be associated with ECM degradation. Hence, we propose that Rab21 may play an important role in *in vivo* tissue invasion and thereby modulate the virulence of the pathogen.

## Materials and Methods

### Organism and culture

*Entamoeba histolytica* trophozoites of HM-1: IMSS cl6 and G3 strain[35] (the G3 strain was generously gifted by Prof. David Mirelman, Department of Biochemistry, Weizmann Institute of Science, Israel) were cultured axenically in BI-S-33 medium supplemented with 15% heat inactivated Adult Bovine Serum (Sigma Aldrich) at 35°C as described previously[54].

### Plasmids

The cloning of Rab21 has been described previously (Pathema ID: EHI_129330, manuscript under review, PNTD-D-14–01300). Briefly, total RNA was extracted using RNA easy kit (Qiagen) and cDNA prepared using the High Capacity RNA to cDNA kit (Applied Biosystems).Rab21 was amplified from the cDNA pool of *Entamoeba histolytica* using the following primer set; forward 5-ATGGAAAACGAATTTAAAGTAGTTTTGTTG-3 and reverse 5-TTAACAACAATCAGATTTTGCTTGACGAGT-3 and cloned into InsaTA cloning vector (Thermo Fisher). It was then further subcloned into the amoebic expression vector pEhExHA using SmaI/XhoI.

For generation of the Rab21 knockdown strain the following primer set was used; forward 5-TTCAGGCCTATGGAAAACGAATTTAAA-3 and reverse 5-TTCGAGCTC AGCTTCTTCTTTTGAAAT-3. Briefly, total RNA was extracted from HM-I: IMSS cl6 strain using RNA Easy kit (Qiagen) and cDNA was prepared using High Capacity RNA to cDNA kit (Applied Biosystems). Further, using standard PCR procedure a 400bp segment of Rab21 was amplified using the above set of primers from the cDNA pool and cloned intopsAP2Gunma using StuI/SacI to generate the gene silencing plasmid psAP2GunmaRab21[55]. The control strain used for the study was G3 transfected with empty psAP2Gunma.

The nucleotide hydrolysis deficient, Rab21CA (Q64L) and the nucleotide binding deficient, Rab21DN (T18N) mutants were generated using Site Directed Mutagenesis strategy (SDM) using the following primer sets; forward 5-GGGATACTGCAGGACTAGAAAAATACCAAGC-3 and reverse 5-GCTTGGTATTTTTCTAGTCCTGCAGTATCCC-3; forward 5-GAAGGAAAAGTTGGAAAGAATTCGATGATATTAAGA-3 and reverse 5-TCTTAATATCATCGAATTCTTTCCAACTTTTCCTTC-3, respectively. pEhExHA Rab21WT was used as the template for the SDM.

The following plasmids pEhExHA, pEhExMyc, pEhExHARab5WT, pEhExMyc Rab7AWT[52], pEhExHA Rab11BWT[53], pEhExHA Rab7BWT[52] and the gene silencing plasmid psAP2Gunma[55]were described previously.

### Generation of amoebic transgenic lines

Logarithmic phase trophozoites (HM1:IMSS for over-expression and G3 strain for gene silencing) were electroporated with the following plasmids; pEhExHA/pEhExMyc (vector control), Rab21WT, Rab21CA, Rab21DN,Rab5WT, Rab7AWT, Rab7BWT, Rab11BWT,psAP2Gunma and psAP2GunmaRab21 using Biorad MX Cell electroporator using the standard protocol.

Briefly, logarithmic phase trophozoites were harvested and washed with chilled 1XPBS, followed by incomplete cytomix buffer (10mM K_2_HPO_4_ /KH_2_PO_4_ (pH 7.6), 120mM KCl, 0.15mM CaCl_2,_ 25mM HEPES (pH 7.4), 2mM EGTA, 5mM MgCl_2_). The washed trophozoites were resuspended in chilled complete cytomix buffer (incomplete cytomix buffer supplemented with 4mM ATP, 10mM reduced glutathione) and 100μg of the respective plasmids were added and cells were electroporated at 500V, and 500μF and immediately transferred to warm complete medium. The transfected trophozoites were selected by adding 4μg/ml G418 (Sigma Aldrich) after 48hrs of transfection. The concentration of G418 was gradually increased to 20μg/ml in the following two weeks. All the transformants were maintained at 20μg/ml of G418 for all the experiments carried out.

For generating the Rab21KD strain, logarithmic phase G3 trophozoites were electroporated with psAP2GunmaRab21.The detailed methodology has been described previously[55].The concentration of G418 was gradually increased to 20μg/ml in the following two weeks. All the transformants were maintained at 20μg/ml of G418.

### Antibodies and reagents

Anti HA (cat. no. sc-7392) and anti Myc (cat. no. sc-40) mouse monoclonal antibodies were purchased from Santa Cruz. Anti Vinculin (hVIN1, cat. no. V9131) mouse monoclonal antibody was purchased from Sigma Aldrich. Alexa Fluor488 anti mouse and Alexa Fluor568 Phalloidin were obtained from Molecular Probes (Invitrogen). Mouse αHgl antibody (3F4) was a generous gift from Dr. William Petri (Department of Microbiology, University of Virginia). Fibronectin from human plasma (cat. no. F2006) was purchased from Sigma Aldrich and collagen type I from rat tail (cat. no. A10483-01) was obtained from Life Technologies. G418 (cat. no. 1720) was purchased from Sigma Aldrich. GalNAc (cat. no. A2795)was purchased Sigma Aldrich. All the chemicals used for the experiments were purchased from Sigma Aldrich.

### Indirect immunofluorescence

Amoeba transformants in logarithmic phase were harvested and transferred to an eight well glass slide and incubated at 37°C water bath for 30 min to let the trophozoites attach to the slide.

Cells were fixed with 4% PFA at room temperature for 15 min, permeabilized with 0.1% tritonX100, blocked with 5% FCS in PBS and stained with anti HA (1:250) or anti Myc (1:250) at room temperature for an hour, followed by Alexa fluorophore conjugated secondary antibodies (1:500) and Alexa568 Phalloidin (1:40) at room temperature for an hour.

The cells were subsequently washed and then mounted in mowiol (mounting medium) and the slides were left overnight at RT for drying. Samples were examined on a Zeiss LSM780 confocal laser scanning microscope using 63x, 1.4 NA oil immersion objective. Images acquired were further quantified (image based quantification) by using Motion Tracking software freely available at the website http://motiontracking.mpi-cbg.de[56,57].

### GalNAc inhibition assay

Amoeba transformants in logarithmic phase were harvested and transferred to an eight well glass slide and incubated at 37°C water bath for 30 min to let the trophozoites attach to the slide. The medium was removed and fresh medium containing different concentrations of GalNAc was added. Maltose at 100mM was included as a negative control. The cells were incubated for an additional hour at 37°C. After an hour, the cells were fixed, permeabilsed, blocked and stained for actin using Alexa568 Phalloidin (1:40) at room temperature for an hour. The samples were mounted in mowiol and dried overnight at RT; they were imaged using Zeiss LSM780. The images obtained were analyzed and quantified using Motion Tracking software.

Similar setup was used for GalNAc inhibition assay with MAb against Hgl except that the cells were incubated with medium containing αHgl 3F4 (1:30) for an hour at 37°C.

### Preparation of fibronectin and collagen coated coverslips

Fibronectin and collagen type I was coated onto an eight well glass slide at a concentration of 100μg/ml under the laminar hood for 1 hr at room temperature. The excess of the FN and collagen type I was aspirated and the wells were washed gently with sterile PBS at RT. The slides were dried under the hood and used for further experiments.

Rab21CA overexpressing cells and vector control were harvested at 400xg, 3min at RT and plated on the coated slides. The cells were further incubated at 37°C for an hour and then fixed, permeabilsed and stained with Alexa568 Phalloidin to visualize actin.

### Fluorescent matrix degradation assay

Fluorescent matrix-coated coverslips were prepared and the assay carried out as described. Briefly, thin layer of Hilyte488 Fibronectin at 100μg/ml concentration (cat.no. FNR02, Cytoskeleton Inc.) was coated on coverslips precoated with 0.01% of poly-L-lysine (cat. no. P8920, Sigma Aldrich) which was cross-linked with 0.5% ice cold glutaraldehyde (cat. no. G5882, Sigma Aldrich) for 15 min at 4°C. The coverslips were incubated in the dark for 3 hours at 37°C. Further, the coverslips were immersed in 5 mg/ml NaBH_4_for 3 min at room temperature. Finally, after a wash and short 10-min incubation in 70% ethanol, coverslips were quenched with complete TYI medium containing 15% adult bovine serum for 1 h at 37°C before cells were plated. Cells were then cultured on ECM-coated coverslips over periods ranging from 36 to 48 h under anaerobic conditions (Gas Pak EZ cat. no. 260683, BD Biosciences).

### Matrigel invasion assay

Matrigel invasion assay was performed essentially as described previously [34]. Briefly, amoeba in the logarithmic growth phase were detached on ice and harvested, suspended in serum free TYI growth medium, and approximately 75,000 cells loaded in the upper chamber of a Transwell migration chamber (8μm pore size, cat. no. 353097, Falcon) coated with 5mg/ml of matrigel (cat. no.354277, Corning). The lower chamber contained growth medium supplemented with 15% adult bovine serum. Transwell plates were incubated at 37°C for 48–50 hrs under anaerobic conditions (Gas Pak EZ, cat. no. 260683, BD Biosciences). Migrated trophozoites attached to the lower chamber wall were detached on ice, harvested, and counted manually using a heamocytometer.

### Sample preparation for SEM analysis

Rab21CA transfomants were plated on different ECM coated surfaces and incubated for 4–6 hrs at 35°C and processed for SEM analysis.

Briefly, log phase trophozoites were harvested and washed with complete medium and then resuspended in warm complete medium containing 15% adult bovine serum. The trophozoites were plated on glass coverslips in a four well plate placed in a BD EZ Gas Pak and incubated at 37°C for an hour for attachment. Further the medium in the wells was replaced with medium containing 2 mg/ml of collagen type I and 1mg/ml of matrigel and trophozoites were incubated for additional 6–8hrs at 37°C in the GasPak. Thereafter, the medium was removed and the cells were briefly washed with warm 0.1M phosphate buffer (pH 7.4) and fixed using 2.5% EM grade gultaraldehyde in 0.1M phosphate buffer (pH 7.4) at 4°C for overnight. Following day, the cells were dehydrated in a graded series of alcohol (25%, 50%, 75%, 95%) for 15min each at RT followed by 100% for 15 min at RT for three times. The samples were then left for drying at RT for 48–56hrs covered with an aluminum foil. The dried samples were sputter coated with gold using Quorum Q150R ES and were examined and photographed with Zeiss ULTRA PLUS field emission scanning electron microscope operating at 5kV.

### Statistical analysis

Unpaired two tailed Student’s t-test was performed using GraphPad Prism5 software. P values from student’s t-test: *P<0.05, **P<0.01 and ***P<0.001.

## Supporting Information

S1 Method qRT PCR. (DOCX)

S2 Method RBC uptake assay. (DOCX)

S1 Fig Vinculin is localized to the amoebic actin dots. Trophozoites stably expressing (A) Rab21CA and (B)Rab21WT were plated on glass and fixed and stained for vinculin and actin using anti Vinculin (hVIN1) and Alexa 568Phalloidin, respectively and imaged using Zeiss LSM 780. z stacks were acquired with z interval of 1μm. A representative slice from the zstack is shown. Scale bar 10μm.
(TIF)

S2 Fig CytochalasinD inhibits the actin dots. Trophozoites stably expressing (A) Rab21CA and (B) Rab21WT were treated with cytochalasin D for 20min at 35°C and fixed and stained for actin with Alexa568 Phalloidin and imaged. Cytochalasin D disrupted the individual actin dots forming long threads of actin at 6μM and 10μM shown with arrows whereas 2μM cytochalasin D was not inhibitory with cells (Rab21CA) forming actin dots shown with the arrow. Scale bar10μm.
(TIF)

S3 Fig Rab21 does not affect erythrophagocytosis. **A.** Trophozoites stably expressing Rab21WT, RAb21CA and Rab21DN were incubated with Cell tracker Red labeled RBCs at 37°C for 5min. Following incubation cells were washed with chilled PBS and finally resuspended in PBS and immediately scanned in a flow cytometer. The representative graphs represent internalized RBCs.**B.**Cellsstably expressing Rab21WT, RAb21CA and Rab21DN were incubated with Cell tracker Red labeled RBCs for 5min at 37°C and immediately washed with warm PBS and fixed, permeablized and stained using anti HA (1:250), followed by secondary anti mouse Alexa488 secondary antibody. The fluorescence and the DIC images were acquired using Ziess ApoTome.2. Scale bar, 10μm.
(TIF)

S4 Fig Rab21CA and Rab21DN over-expression alters gross surface topology of the trophozoites. Scanning electron micrograph of vector control (pEhExHA), Rab21CA and Rab21DN cells plated on glass; Control cells exhibit prominent membrane protrusive structures whereas the mutants are devoid of them. Rab21CA over expressing cells are spread out and extended as compared to Rab21DN which are rounded up. Left panel shows a lower magnification electron micrograph (1000X) and right panel shows a higher magnification electron micrograph (8000X). Scale bars 20μm (left panel) and 2μm (right panel).
(TIF)

S5 Fig Generation of Rab21 knock down strain. Relative expression ofRab21 under standard axenicculture conditions in vector (psAP2Gunma) and Rab21KD strain.
(TIF)
