# Supplementary material for: Small GTPase Rab21 Mediates Fibronectin Induced Actin Reorganization in Entamoeba histolytica: Implications in Pathogen Invasion
Source: PLoS Pathog. 2015 Mar 2;11(3):e1004666. doi: 10.1371/journal.ppat.1004666 (PMC4346268; doi:10.1371/journal.ppat.1004666)
Supplement: S1 Method — (DOCX) [file ppat.1004666.s001.docx]

**S1 Method. qRT PCR**

Total RNA was extracted from G3 strain transfected with empty vector (psAP2Gunma) and

psAP2GunmaRab21 (Rab21KD) using RNA easy kit (Cat. 74104, Qiagen, Limburg, Netherlands) andcDNA prepared using the High Capacity RNA to cDNA kit (Cat. 4387406, Life Technologies,Inc, Carlsbad, CA) qRT PCR was performed on the resulting cDNA template using the following primer set for Rab21; forward 5’-ACTCCTGAAGAAGGTCCTCGT-3’ and reverse 5’-CGTTGGAAACTTCTTGGTTCAGT-3’ and SYBR Green in an Applied Biosystems 7300 Real Time PCR system. RNA Polymerase and actin were used as internal controls.
