# Supplementary material for: Small GTPase Rab21 Mediates Fibronectin Induced Actin Reorganization in Entamoeba histolytica: Implications in Pathogen Invasion
Source: PLoS Pathog. 2015 Mar 2;11(3):e1004666. doi: 10.1371/journal.ppat.1004666 (PMC4346268; doi:10.1371/journal.ppat.1004666)
Supplement: S2 Method — (DOCX) [file ppat.1004666.s002.docx]

**S2 Method. RBC uptake assay**

Red blood cells were harvested and labeled with 20μM of CellTracker Red CMPTX (Cat no. C34552, Life Technologies) for an hour at 37°C in dark. The RBCs were subsequently washed with 1%BSA in PBS and finally resuspended in warm BI medium. The logarithmic phase amebic trophozoites expressing Rab21 WT, CA and DN mutants were incubated on ice and detached. Trophozoites were harvested and resuspended in warm BI medium and transferred to a 6 well culture plate. Labeled RBCs were added to each well and incubated for 5 min at 37°C, immediately after which the cells were washed with chilled PBS, fixed with 4% PFA and harvested and analysed using BD-FACS Aria III (Becton Dickinson, East Rutherford, NJ).

For immunofluorescence, the cell tracker labeled RBCs were incubated with Rab21WT and CA, DN mutants for 5min at 37°C in an 8 well slide and further cells were washed, fixed and stained with mouse anti HA (1:250) for an hour at RT, followed by secondary anti mouse Alexa488 (1:500) and analyzed using on Zeiss ApoTome.2 mounted on an AXIO ImagerM2 using a 63x, 1.4 NA oil immersion objective.
