# Supplementary figures and images for: Small GTPase Rab21 Mediates Fibronectin Induced Actin Reorganization in Entamoeba histolytica: Implications in Pathogen Invasion
Source: PLoS Pathog. 2015 Mar 2;11(3):e1004666. doi: 10.1371/journal.ppat.1004666 (PMC4346268; doi:10.1371/journal.ppat.1004666)

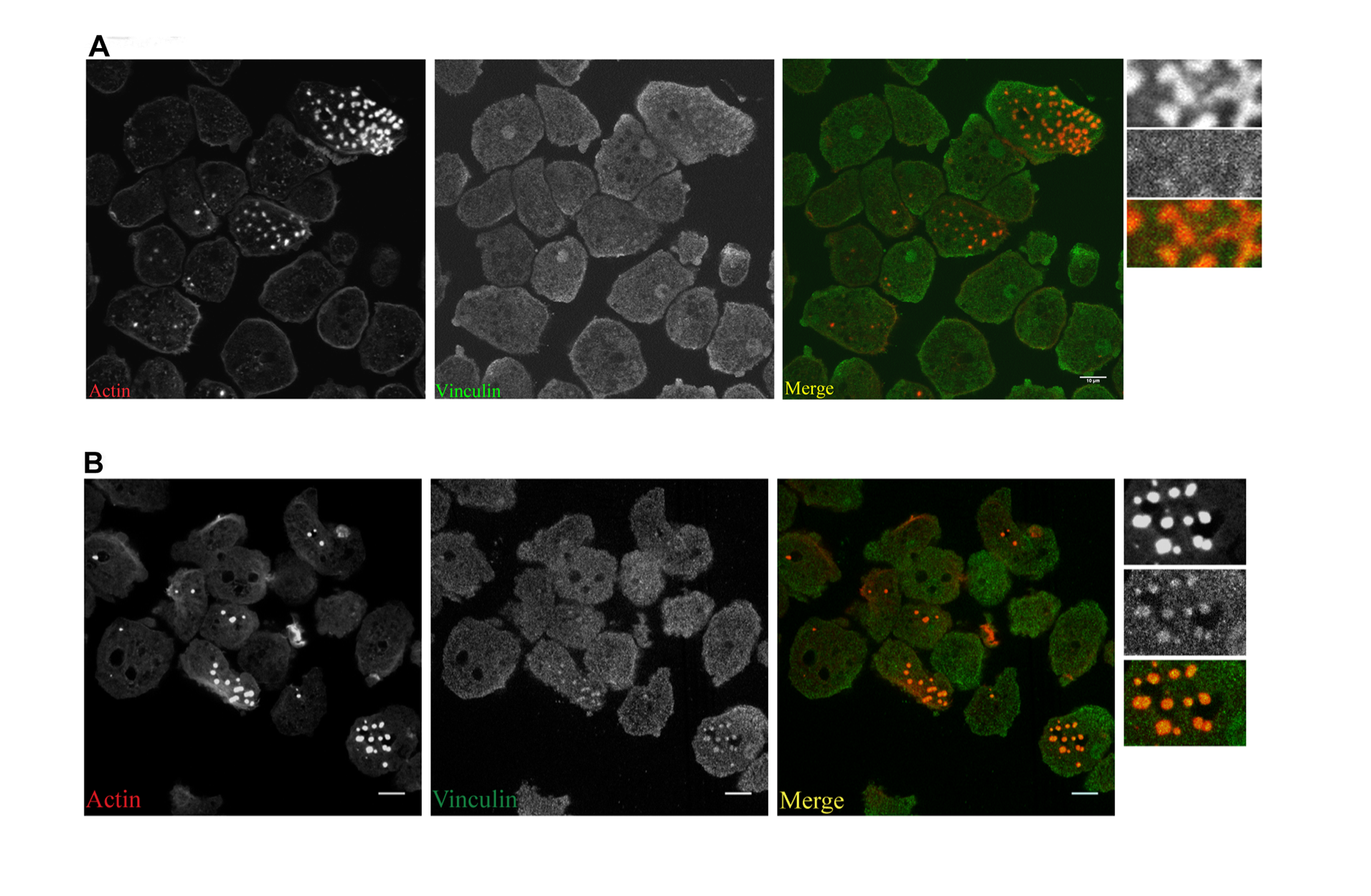

Supplement: S1 Fig — Trophozoites stably expressing (A) Rab21CA and (B)Rab21WT were plated on glass and fixed and stained for vinculin and actin using anti Vinculin (hVIN1) and Alexa 568Phalloidin, respectively and imaged using Zeiss LSM 780. z stacks were acquired with z interval of 1μm. A representative slice from the zstack is shown. Scale bar 10μm. (TIF) [file ppat.1004666.s003.tif]

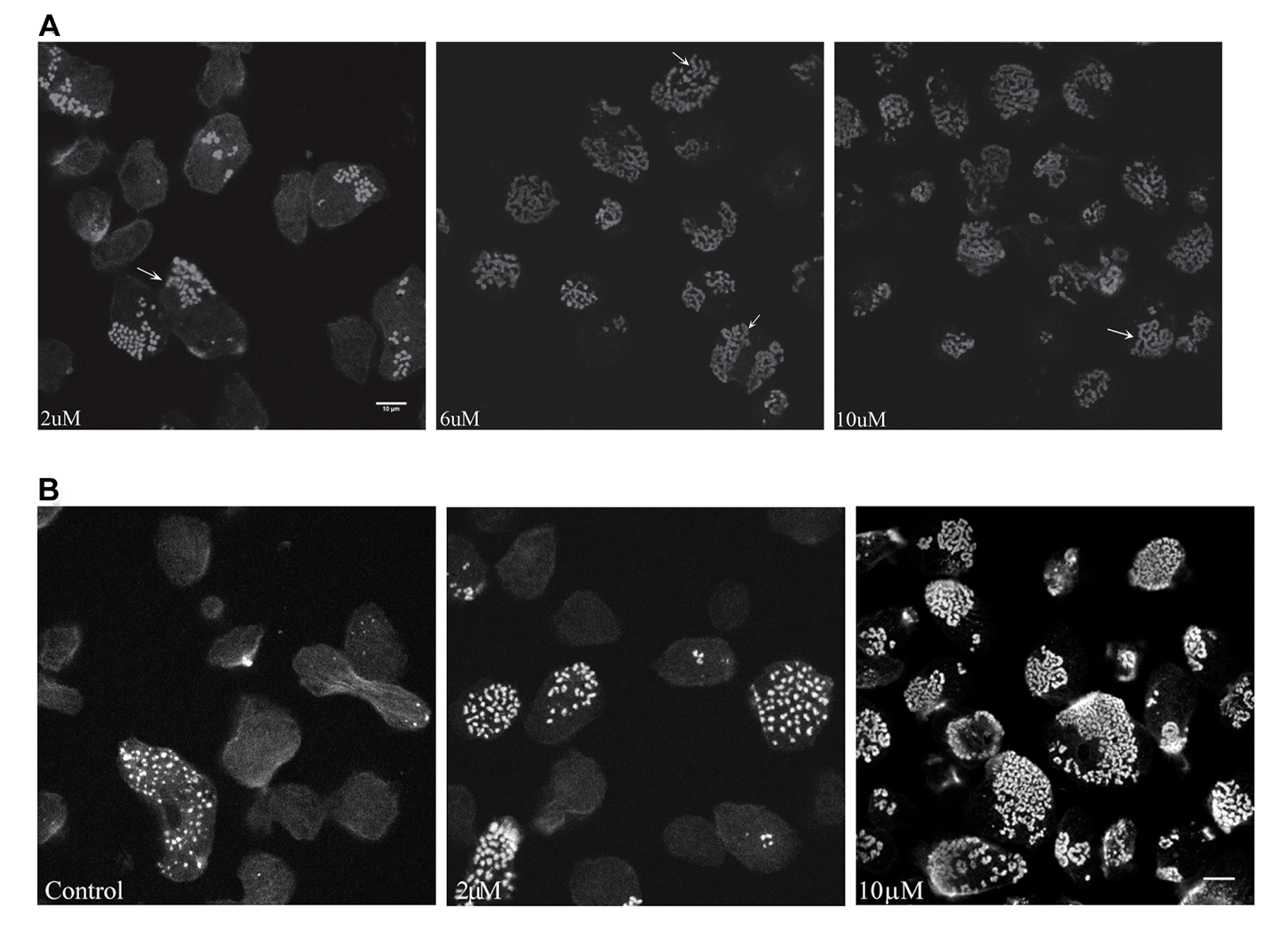

Supplement: S2 Fig — Trophozoites stably expressing (A) Rab21CA and (B) Rab21WT were treated with cytochalasin D for 20min at 35°C and fixed and stained for actin with Alexa568 Phalloidin and imaged. Cytochalasin D disrupted the individual actin dots forming long threads of actin at 6μM and 10μM shown with arrows whereas 2μM cytochalasin D was not inhibitory with cells (Rab21CA) forming actin dots shown with the arrow. Scale bar10μm. (TIF) [file ppat.1004666.s004.tif]

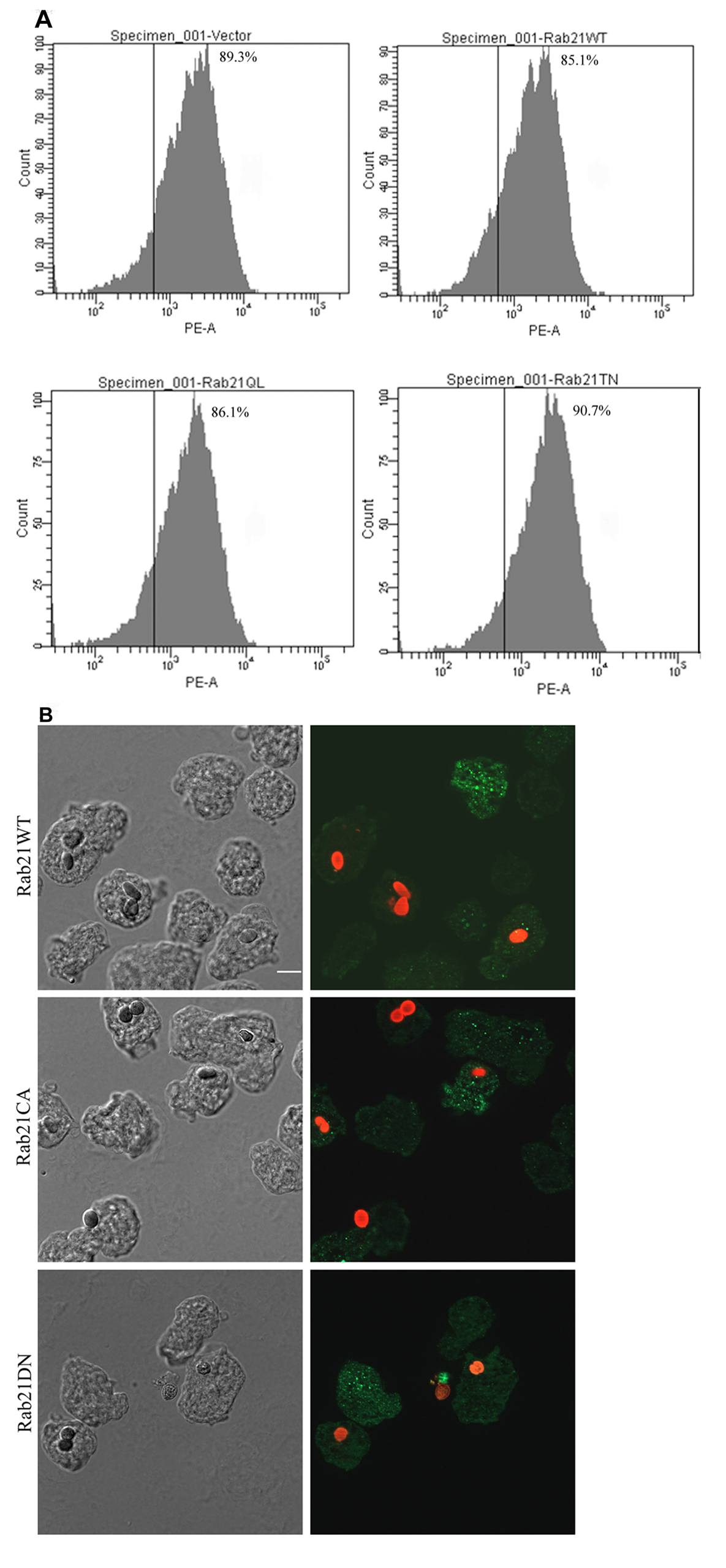

Supplement: S3 Fig — A. Trophozoites stably expressing Rab21WT, RAb21CA and Rab21DN were incubated with Cell tracker Red labeled RBCs at 37°C for 5min. Following incubation cells were washed with chilled PBS and finally resuspended in PBS and immediately scanned in a flow cytometer. The representative graphs represent internalized RBCs.B.Cellsstably expressing Rab21WT, RAb21CA and Rab21DN were incubated with Cell tracker Red labeled RBCs for 5min at 37°C and immediately washed with warm PBS and fixed, permeablized and stained using anti HA (1:250), followed by secondary anti mouse Alexa488 secondary antibody. The fluorescence and the DIC images were acquired using Ziess ApoTome.2. Scale bar, 10μm. (TIF) [file ppat.1004666.s005.tif]

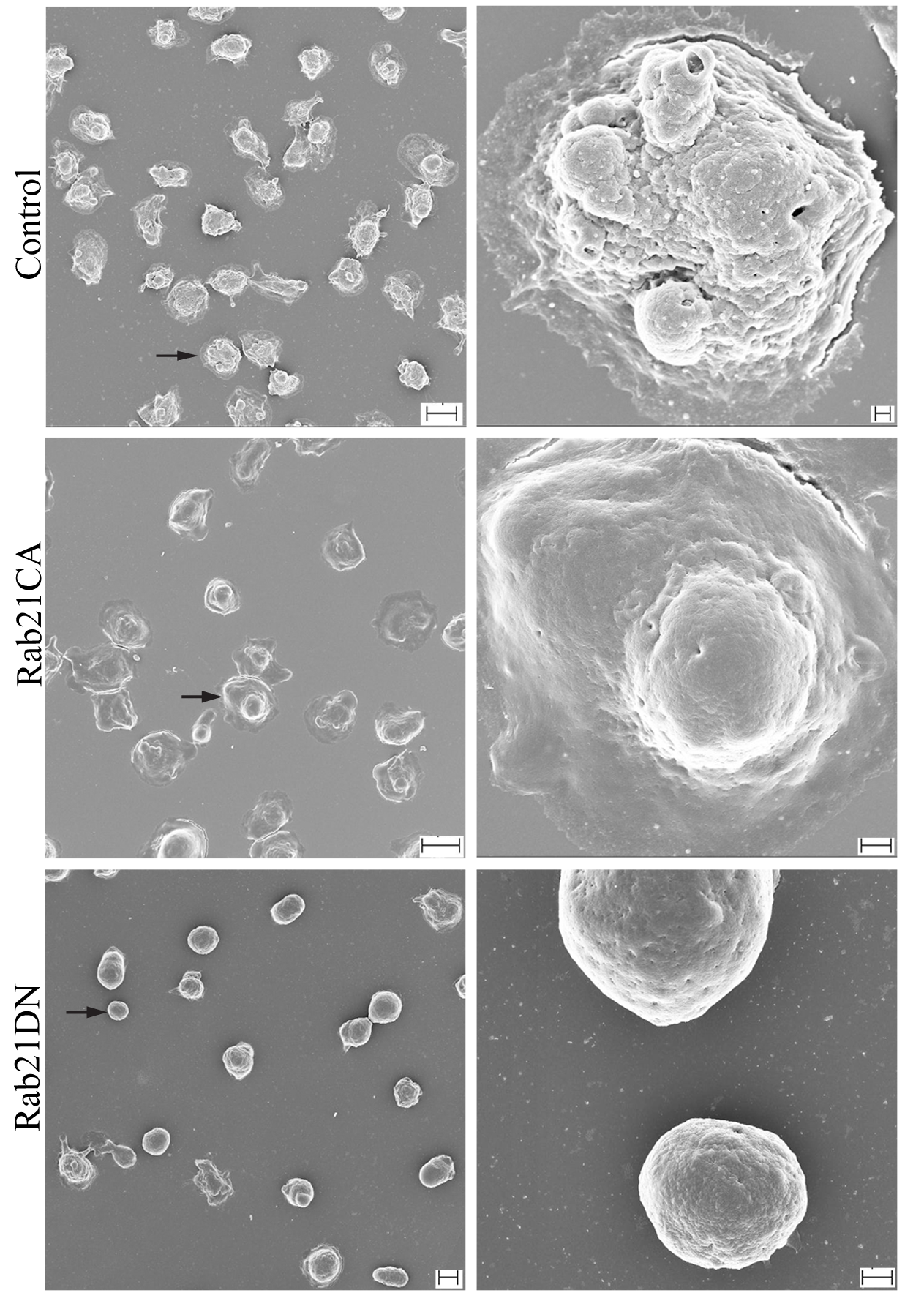

Supplement: S4 Fig — Scanning electron micrograph of vector control (pEhExHA), Rab21CA and Rab21DN cells plated on glass; Control cells exhibit prominent membrane protrusive structures whereas the mutants are devoid of them. Rab21CA over expressing cells are spread out and extended as compared to Rab21DN which are rounded up. Left panel shows a lower magnification electron micrograph (1000X) and right panel shows a higher magnification electron micrograph (8000X). Scale bars 20μm (left panel) and 2μm (right panel). (TIF) [file ppat.1004666.s006.tif]

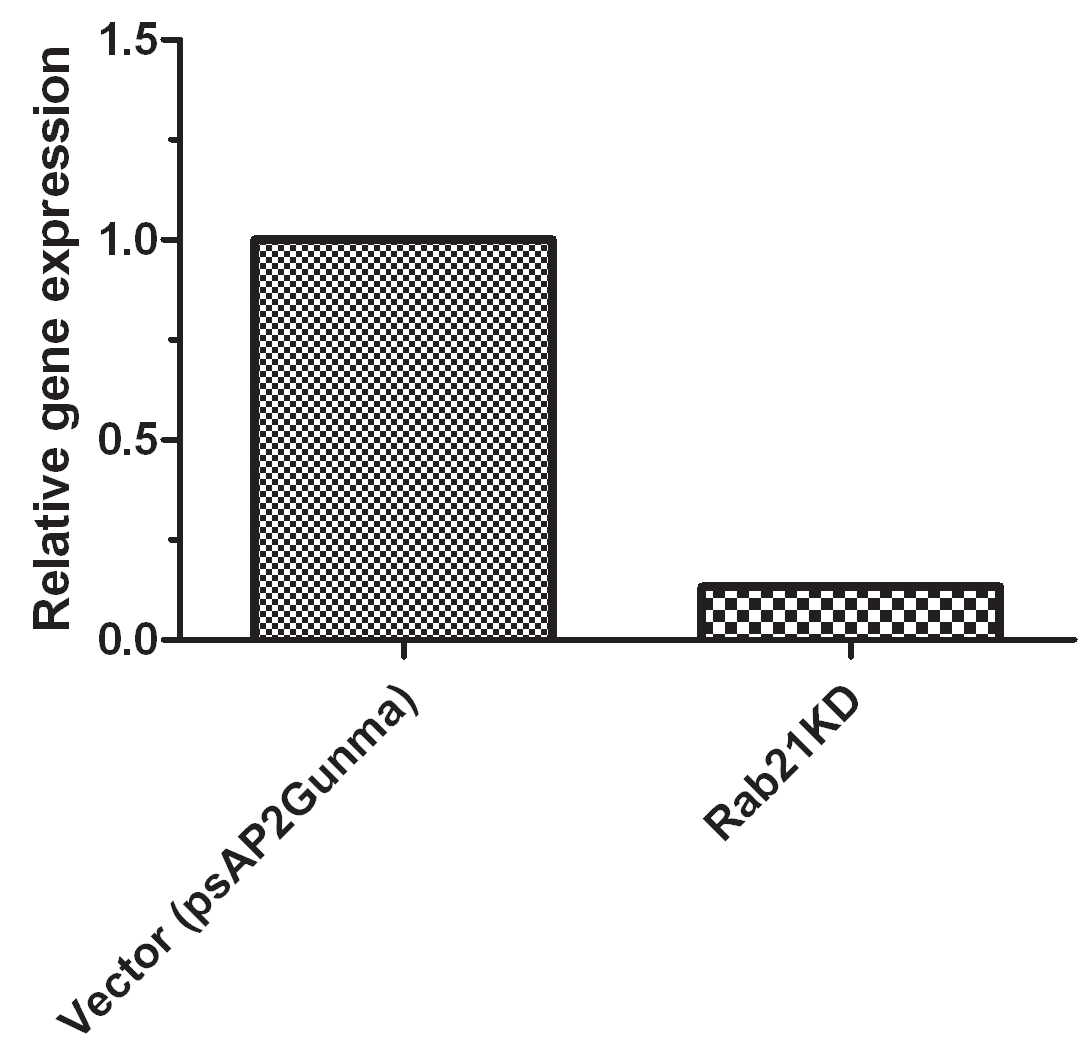

Supplement: S5 Fig — Relative expression ofRab21 under standard axenicculture conditions in vector (psAP2Gunma) and Rab21KD strain. (TIF) [file ppat.1004666.s007.tif]
